# Green synthesis of calcium oxide nanocatalyst and application in transesterification of waste cooking oil

**DOI:** 10.1186/s40643-025-00879-4

**Published:** 2025-06-02

**Authors:** Rajni Garg, Rishav Garg, Nnabuk Okon Eddy, Mukhtar Iderawumi Abdulraheem, Oluwadamilola Oluwatoyin Hazzan, Gholaremza Abdi

**Affiliations:** 1 Department of Applied Sciences, Galgotias College of Engineering & Technology, Greater Noida, 201310 India; 2 Department of Civil Engineering, Galgotias College of Engineering & Technology, Greater Noida, 201310 India; 3 Department of Pure and Industrial Chemistry, University of Nigeria, Nsukka, Enugu State Nigeria; 4 Department of Electrical Engineering, Henan Agricultural University, Zhengzhou, 450002 China; 5 Institute of Urban Environment, Chinese Academy of Sciences, Xiamen, China; 6 Department of Biotechnology, Persian Gulf Research Institute, Persian Gulf University, Bushehr, 75169 Iran

**Keywords:** Green synthesis, Nano-catalyst, Waste cooking oil, Transesterification, Biodiesel

## Abstract

This study aims to synthesize calcium oxide nanoparticles by employing green synthetic methods and explore their potential as nano-catalyst based upon the utilization of waste into a value-based product. Waste orange peel extract has been utilized as a reducing medium. The reaction was optimized by varying the reactants’ molar ratio to obtain calcium carbonate microparticles that were calcined to obtain calcium oxide nanoparticles with a particle size ranging from 70 to 100 nm. Various spectrochemical techniques analyzed the composition and morphology of the nano-catalyst. The nano-catalyst was further exploited in the one-pot transesterification of waste cooking oil. The biodiesel was analyzed for the presence of methyl ester groups by FTIR and GCMS analysis. The impact of varying reaction constraints, including temperature, contact time, nano-catalyst concentration, and methanol-oil molar ratio, were critically analyzed to optimize biodiesel yield. The study provided an economical and environmentally benign technique to successfully synthesize calcium oxide nano-catalyst to obtain biodiesel with 93.4% yield and effective waste minimization.

**Graphical Abstract:**

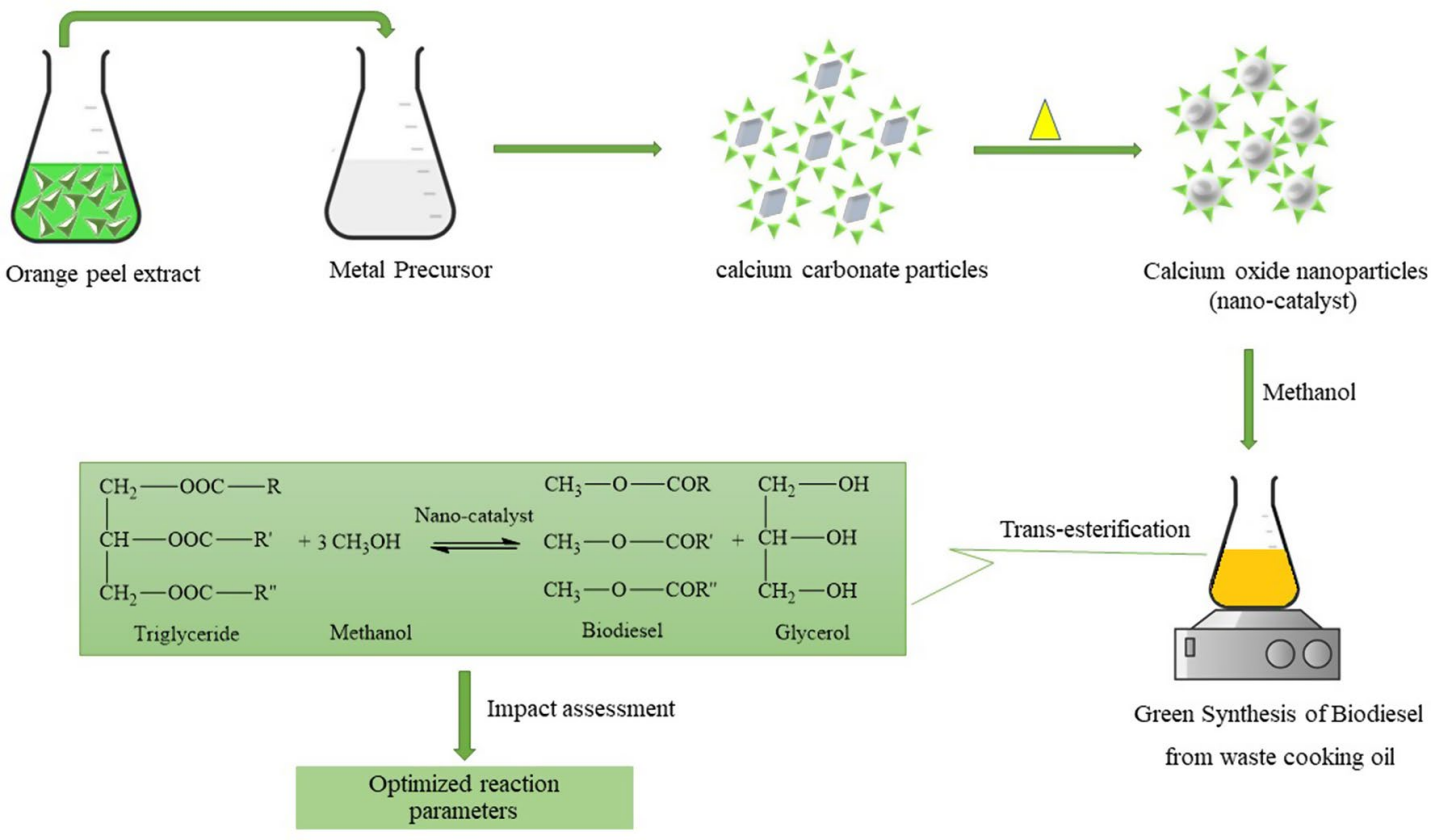

## Introduction

Waste cooking oil (WCO) is usually a by-product produced in huge amounts while cooking in various food joints. It is generally consumed for producing soap and animal feed or disposed of improperly (Erchamo et al. 2021). Thus, its effective management is still a significant challenge, leading to contamination and environmental concerns due to disposal issues (Ullah et al. 2014). Its usage for obtaining biodiesel is a viable option in the current scenario of sustainable development. Biodiesel is a non-harmful, biodegradable, sustainable, and alternative fuel for efficiently reducing greenhouse gas emissions. Alternative fuels are gaining increased importance due to the environmental impact of depleting petroleum reserves and automobile exhaust gases (Kirkok et al. 2021). Manufacturing biodiesel from waste materials is a more cost-effective and efficient alternative fuel production method than other methods (Ulakpa et al. 2024).

The transesterification of oil can proceed as homogenous or heterogeneous catalysis generating a blend of fatty acid methyl esters (FAME). Although the production by homogenous base catalysis can provide high FAME yields under mild reaction conditions, it is still not preferred because of the associated disadvantages (Sarkar et al. 2024). First is the necessity of the regeneration of the homogenous catalyst (mineral acids/bases) because the catalyst is consumed during the undesired saponification reaction (Meena Devi et al. 2015). Further catalyst separation is a difficult process that necessitates acquiring additional equipment resulting in increased manufacturing expenses (Mumtaz et al. 2012). Due to these drawbacks, using heterogeneous catalysts (base earth metal oxides, hydrotalcite, base-doped alumina, and base zeolites) is the better option for biodiesel production as these can be easily separated, and undesired saponification reactions are avoided (Ruatpuia et al. 2023). The employment of solid catalysts viz. tin(II) oxide (Nabihah-Fauzi et al. 2020), titania (Hossain et al. 2019), copper oxide (Suresh et al. 2021), magnesium oxide (Navas et al. 2020), and calcium oxide (Gandhi et al. 2021), also lower the production cost because these catalyst can be recycled and reused (Erchamo et al. 2021).

Calcium oxide (CaO) is amongst the most significant metal oxides, and its biocompatibility, low cost, and ease of manufacture have fascinated researchers for biomimetic and other technology applications (Eddy and Garg 2021). It is used as a catalyst (Joshi et al. 2015), a sequestration and remediation agent for pollutants (Thakur et al. 2022), a purification agent for hot gases (Nuryantini et al. 2019), a doping agent in refractories, and a desulfurization agent for flue gas (Bano and Pillai 2020). Among the various catalysts adopted for biodiesel production, calcium oxide has shown a good ability to carry out transesterification reactions effectively. This can be attributed to factors viz. reusability, moisture tolerance, immiscibility with methanol, and low cost, as it can be obtained from green techniques (Purandaradas et al. 2018). In particular, calcium oxide nanoparticles are an effective option, especially when feedstock has a lower free fatty acid content, as in the case of low-quality waste or used oils.

Nanotechnology involves designing, manipulating, manufacturing, and applying materials at a nanoscale scale. Due to their enhanced electrical, optical, and chemical properties compared to their bulk counterpart and potential applications, nanoparticles have been the primary focus of study (Ahmad et al. 2024). Metal (Au, Ag, Fe, and Pt) and metal oxide (CaO, ZnO, CuO, TiO, NiO, and Fe_3_O_4_) nanoparticles have been employed to boost applications in industrial, pharmaceutical and agricultural sectors because of their size-dependent features (Sharma et al. 2020). CaO nanoparticles have been extensively explored for raw and waste cooking oil transesterification (Basumatary et al. 2023). Various conventional physical and chemical techniques can be used to obtain nanoparticles. Still, with the advent of green chemistry, researchers prefer green synthetic techniques involving the use of plant extracts (Ahmad et al. 2024), micro-organisms (Bahrulolum et al. 2021), and biomolecules such as cellulose (Abdellatif et al. 2021), enzymes (Dadi et al. 2021) etc. Plant extracts are considered a promising reducing medium as their extant phytochemicals perform as reducing, nucleating, and stabilizing agents. Literature reports the use of the leaf extract of *Mentha piperita* (Ijaz et al. 2017), *Moringa oleifera* (Jadhav et al. 2022), *Morus nigra* (Nasir et al. 2024), *Mentha arvensis* (Shanmuganathan et al. 2023), *Acacia Arabica* (Veeramani et al. 2024), *Carica papaya* (B et al. 2020) and *Ocimum sanctum* (Asha et al. 2024) as well as fruit extract of *Citrullus colocynthis* (Mazher et al. 2023), seed extract of *Linum usitatissimum* (Tabrizi Hafez Moghaddas et al. 2024) and peel extract of *Punica granatum* (Ahmad et al. 2024) for synthesis of for CaO nanoparticles.

Among various phyto extracts, the orange extract has been explored to a greater extent because of its functionalities such as limonoids, flavonoids, polymethoxylated flavones, coumarins, hydroxycinnamates, and psoralens (Manthey and Grohmann 2001). Although many researchers have synthesized CaO nanoparticles using duck eggshells (Nuryantini et al. 2019), Chicken eggshells (El-sherif et al. 2023), gastropod shells (Oladoja et al. 2012), and cockle shells (Jalu et al. 2021), yet orange peel extract has not been explored for the same, to the best of our knowledge. This study aims to utilize orange peel extract as a reducing medium for the green synthesis of calcium carbonate microparticles that have been further used as a precursor for nano-calcium oxide for potential use as a heterogeneous nano-catalyst. The nano-catalyst has been used for transesterification of waste cooking oil to obtain biodiesel. Thus, the study provides an eco-friendly and economical process for waste valorization to obtain value-added products.

## Materials and methods

### Materials

All chemical compounds were acquired from Merck and utilized as such. Waste cooking (Soyabean) oil was collected from a local restaurant just after third frying cycle. The oil was filtered and heated at 120^o^C to remove insoluble properties and moisture. The acid value of the oil was found to be very low (1.57 mg KOH/g) after standard analysis (Sarkar et al. 2024). Orange peels were obtained from a neighborhood fruit juice store. All solutions were prepared using triple distilled water.

### Preparation of aqueous extract of orange Peel

Fresh orange peels were cleaned using distilled water to eliminate dust and impurities. 25 g of clean peels were chopped and boiled (80 °C for 30 min) in 150 mL triple distilled water. Whatman filter paper (No. 1) was used for filtering the solution after it had been cooled to room temperature to obtain the peel extract.

### Synthesis and characterization of nano-catalyst

During phase-I, 50 mM aqueous Na_2_CO_3_ and CaCl_2_ prepared in two separate beakers were added to the peel extract at varying volume proportions. The white precipitates of calcium carbonate were immediately obtained in the reaction mixture. However, the best yield was obtained at a volume proportion of 5:4:1 for Na_2_CO_3_, CaCl_2,_ and peel extract. The precipitates were accumulated by centrifugation at 4000 rpm, followed by washing with triple distilled water and ethanol. During phase-II, the precipitates were vacuum-dried and calcined at 850^o^C for 12 h to remove CO_2_ produced during the conversion of CaCO_3_ to CaO. The calcined product was then stored in a desiccator for subsequent use.

The precipitates of CaCO_3_ and CaO nano-catalyst were characterized using a scanning electron microscope (Model JEOL JSM6100) with an accelerating voltage of 20 KV after super coating within a layer of gold. The crystalline phase was determined by X-Ray powder diffraction (Model PANalyticalX’Pert Pro). FTIR spectroscopic measurements were carried out using FT-IR Spectrometer (Model Perkin Elmer Spectrum 400).

### Synthesis and characterization of biodiesel

The nano-catalyst was pretreated with methanol before employing in the transesterification reaction. The oil sample was pretreated with anhydrous sodium sulfate in a round bottom reactor (1000 ml) attached to an overhead mechanical stirrer, a reflux condenser, and a thermostat. The reactions were performed with a variation of the reaction temperature (50-65^o^C), reaction time (2.5–5.0 h.), catalyst concentration (0.5-2%), and the methanol: oil molar ratio (3:1–15:1). The system was cooled, and the nano-catalyst was retrieved with centrifugation, splashed with n-hexane and dried at 110^o^C for 2 h. for reuse. Biodiesel was obtained after separating glycerol and removing unreacted methanol. The standard method computed the final yield of biodiesel (Rahman et al. 2019). The sample was analyzed by FT-IR Spectrometer (Model Perkin Elmer Spectrum 400) and GC-MS (Model THERMO Scientific Trace 1300GC) techniques. The physicochemical properties of biodiesel were analysed by standard methods.

## Results and discussion

In this study, orange peel extract has been explored as a reducing medium for the green synthesis of calcium carbonate transformed to calcium oxide by calcination for potential use as a nano-catalyst, as discussed ahead.

### A mechanism for the formation of nano-catalyst

The biomineralization process is a natural phenomenon used by many organisms for direct nucleation and growth of calcium carbonate to form skeletons or shells (seashells, exoskeletons of arthropods and corals, etc.). Calcium carbonate is found to exist mainly in three crystalline polymorphs: aragonite, calcite, and vaterite (Lin et al. 2020). Out of these, vaterite is the thermodynamically most unstable, while calcite is the most stable polymorph. Calcite has a rhombohedral crystalline structure found in sedimentary limestone and rocks. Argonite has an orthorhombic crystalline structure and is the main constituent of calcareous organisms’ skeletal systems. Vaterite has a hexagonal spherical or irregular structure and occurs in the endoskeleton of some organisms. However, vaterite polymorph can be quickly transformed into stable calcite form using particular solvents (Ghavamifar et al. 2023). Due to the great biological significance of these polymorphs, scientists are investigating the methods and mechanisms for crystallization. Water-soluble additives, including polymers, biomolecules, and simple metal ions, initiate the crystallization and polymorph transition (El-sherif et al. 2023).

**Fig. 1:**
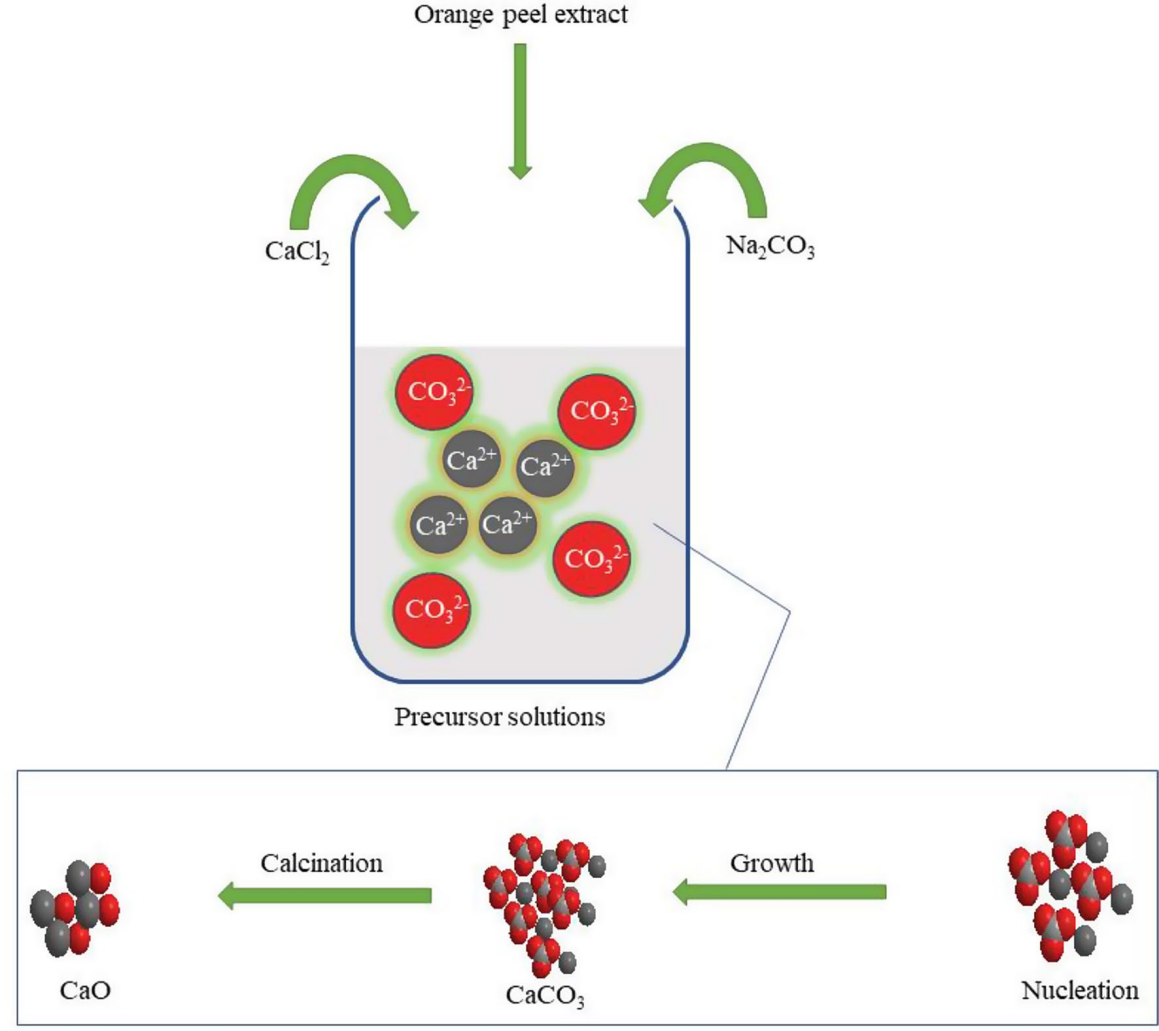
The mechanism for the synthesis of nano-catalyst

Many researchers have used biomolecules such as polysaccharides to induce calcium carbonate crystallization. Polysaccharides are highly bioactive compounds and contain hydroxyl, carboxy, and keto functional moieties, etc. (Eddy et al. 2023). These functional moieties are supposed to initiate nucleation by binding calcium ions or through adsorption on the crystal surface and facilitate the overall crystallization process (Fig. 1). During green synthesis employing fruit extract, the formation of calcium carbonate particles is supported by soluble polysaccharides in the extract (Garg et al. 2020). The polysaccharides contain a negatively charged hydroxy group that can strongly react with Ca^2+^ ions resulting in their accumulation. As supersaturation is achieved, crystallization gets initiated at the site of Ca^2+^ accumulation leading to the aggregation of particles. Thus, the presence of polysaccharides helps form nucleation sites at which the fine-sized particles of calcium carbonate start deposition. The polyhydroxy moieties in the extract also bind with these particles and provide capping and stabilization to these particles that attain rhombohedral or sheaf-like structures (Mistretta et al. 2021). During calcination of calcium carbonate at a suitable temperature, decomposition leads to the formation of calcium oxide and the release of carbon dioxide (Ogoko et al. 2024).

### Characterization of nano-catalyst

Various spectrochemical analyses have been used to analyze the precipitates obtained in phase-I and the nano-catalyst obtained in phase-II, and the results have been discussed ahead.

**Fig. 2:**
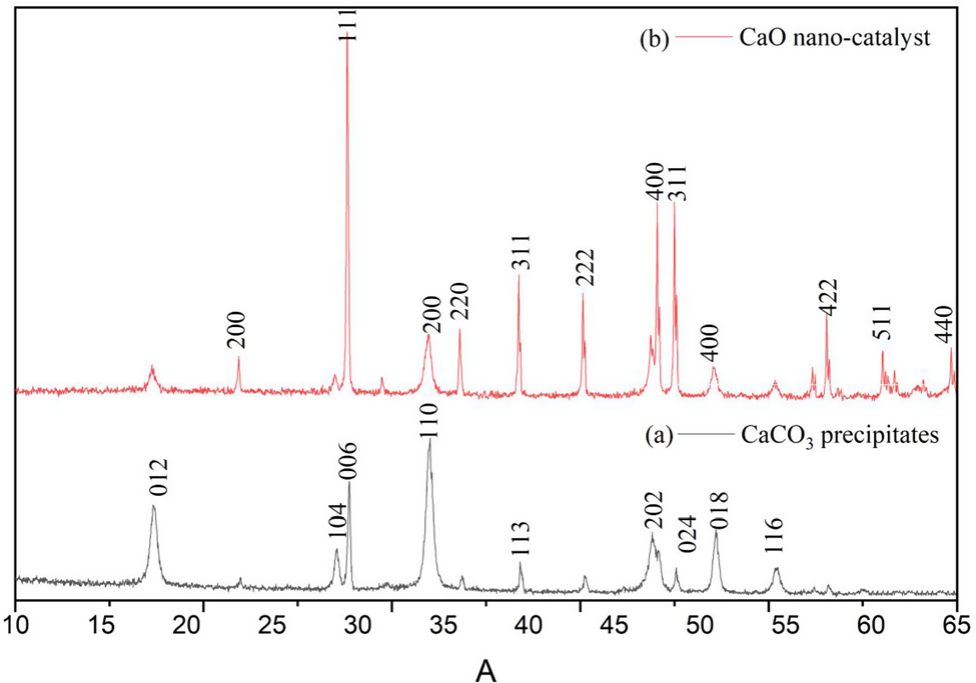
XRD patterns for precipitated calcium carbonate (**a**) and nano-catalyst (**b**)

XRD analysis is very important to ascertain the crystalline structure and phase of the particles (Ulakpa et al. 2024). The crystalline phase of the CaCO_3_ precipitates and the nano-catalyst were investigated using X-ray diffraction examination. Figure 2a demonstrates the XRD pattern of precipitates obtained in the green synthesis using peel extract. The high-intensity diffraction peaks confirm the crystalline polymorph phases of calcium carbonate. The uncalcined precipitates are mainly composed of calcite phases of CaCO_3_ (JCPDS: 05-0586) with diffraction peaks at 2θ equal to 18.17^o^ (012), 29.511^o^ (104), 29.02^o^ (006), 34.19^o^ (110), 39.52^o^ (113), 47.17^o^ (202), 50.95^o^ (018), 54.56^o^ (116), and 48.65^o^ (024) (Fig. 2a). Additionally, no peak associated with any other compound was evident in the XRD pattern, supporting the purity of the product (Erchamo et al. 2021). The X-ray diffraction pattern with characteristic peaks for the obtained nano-catalyst has been represented in Fig. 2b. The narrow and intense peaks obtained in the patterns were attributed to the crystalline structure of CaO (JCPDS: 05-0586) (Erchamo et al. 2021). The calcined product showed characteristic peaks for CaO at 2θ equal to 21.74^o^ (200), 29.40^o^ (111), 34.10^o^ (200), 35.96^o^ (220), 39.40^o^ (311), 43.16^o^ (222), 47.47^o^ (400), 48.51^o^ (311), 50.81^o^ (400), 56.56^o^ (422), 60.71^o^ (511), and 64.67^o^ (440), (Fig. 2b) corroborating the change of CaCO_3_ to CaO after calcination (Fayyazi et al. 2018).

The crystallite size of the particles was obtained from Scherrer’s equation (Eq. 1) relating the crystallite size (*d*_*x*_) to the crystallite-shape factor (*k*), wavelength of the X-ray (λ), full width at half maximum (FWHM), and angle of diffraction ($\:\theta\:)$:

1 $$\:{d}_{x}=\:\frac{k\lambda}{FWHM*cos\theta\:}$$

The average crystallite size of CaCO_3_ was obtained as 25.14 nm (Table 1). Earlier studies have also reported the green synthesis of CaCO_3_ with low crystallite size (Ghiasi and Malekzadeh 2012). On the other hand, the average crystalline size of CaO nano-catalyst was obtained as 24.57 nm.

**Table 1:** Crsytalographic parameters for CaCO_3_ preccipitates and CaO nano-catalyst

2ϴ (^o^)	FWHM	d_x_ (nm)	d_ϴ_ (Å)	Reference	(hkl)	JCPDS Card*	Micro strain (*10^− 3^)	Phase
CaO nano-catalyst								
21.74	0.53	14.62	4.09	4.09	200.00	37-1497	12.13	CaO
29.40	0.31	24.95	3.04	3.04	111.00	37-1497	5.12	CaO
34.10	0.19	40.84	2.63	2.63	200.00	37-1497	2.65	CaO
35.96	0.32	23.40	2.50	2.50	220.00	37-1497	4.34	CaO
39.40	0.52	14.52	2.29	2.29	311.00	37-1497	6.28	CaO
43.16	0.20	37.71	2.09	2.10	222.00	37-1497	2.16	CaO
47.47	0.19	39.13	1.91	1.92	400.00	37-1497	1.84	CaO
48.51	0.21	34.31	1.88	1.88	311.00	37-1497	2.04	CaO
50.81	0.78	9.23	1.80	1.80	400.00	37-1497	7.14	CaO
56.56	0.27	26.16	1.63	1.63	422.00	37-1497	2.17	CaO
60.71	0.41	16.92	1.52	1.52	511.00	37-1497	3.02	CaO
64.67	0.51	13.06	1.44	1.44	440.00	37-1497	3.54	CaO
CaCO_3_ precipitates								
18.17	0.59	13.40	4.88	4.86	12.00	05-0586	15.97	Calcite
29.51	0.34	22.51	3.03	3.03	104.00	05-0586	5.66	Calcite
29.02	0.19	40.79	3.07	3.09	6.00	05-0586	3.18	Calcite
34.19	0.51	14.79	2.62	2.59	110.00	05-0586	7.29	Calcite
39.52	0.17	44.04	2.28	2.28	113.00	05-0586	2.06	Calcite
47.17	0.60	12.04	1.93	1.92	202.00	05-0586	6.04	Calcite
50.95	0.19	38.33	1.79	1.79	18.00	05-0586	1.71	Calcite
54.56	0.34	20.74	1.68	1.68	116.00	05-0586	2.88	Calcite
48.65	0.37	19.59	1.87	1.87	24.00	05-0586	3.57	Calcite

*JCPDS- Joint Committee on Powder Diffraction Standards

The interplanar distance was calculated from the Bragg equation (Eq. 2)(Sahadat Hossain et al. 2023):

2 $$\:{d}_{\theta\:}=\:\frac{n\lambda}{sin\theta}$$

Microstrain is a measure of structural disorder, distortion, or strain resulting in the distortion or imperfection of a crystal. The microstrain parameter (*ε*) was computed through Eq. 3 (Prasad 2016):

3 $$\:\epsilon=\:\frac{FWHM}{4*tan\theta\:}$$

The microstrain in CaCO_3_ varied from 1.71 × 10^− 3^ to 15.97 × 10^− 3^ indicating very less crystallinity imperfection calcite phases. The microstrain was further found to decrease for CaO nano-catalyst with a range varying from 1.84 × 10^− 3^ to 12.12 × 10^− 3^ (Table 1) confirming their comparatively better crystallinity index (Sahadat Hossain et al. 2023).

Fourier transform infrared spectroscopy (FTIR) provides information about the functional groups present in the nanoparticles and the plant extract. FTIR spectra reveal the characteristic vibrational modes of CaCO3, confirming its presence. Furthermore, FTIR can identify the functional groups from the plant extract that are involved in the reduction and capping processes. This analysis provides valuable insights into the mechanisms by which plant components interact with Ca²⁺ ions and influence the synthesis process (Basumatary et al. 2023).

**Fig. 3:**
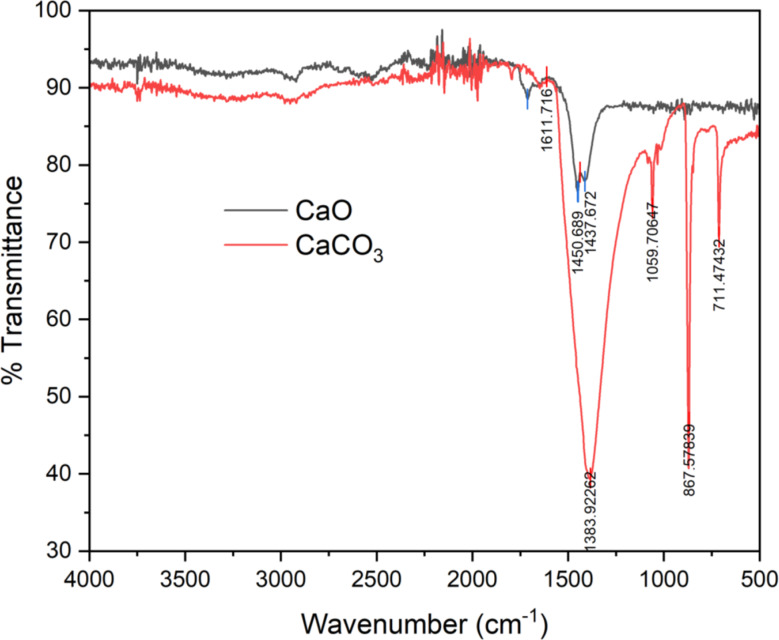
FTIR spectra for precipitated calcium carbonate and nano-catalyst

The uncalcined precipitates of calcium carbonate and the obtained nano-catalyst were characterized using FTIR analysis. The characteristic peaks of calcium carbonate were obtained in the FTIR spectrum and illustrated in Fig. 3. These findings were confirmed with already published in the literature (Erchamo et al. 2021) and provided evidence for the prevalence of calcite phase in the CaCO_3_ precipitates. The prominent sharp peaks at 1059.71 and 711.74 cm^-1^ corresponded to the calcite phase (Hangun-Balkir 2016), whereas the asymmetric stretching of CO_3_^2-^ ions was ascribed to a profound band at 1383.92 cm^-1^ (Garg et al. 2020). A sharp peak around 867.57 cm^-1^ was ascribed to CO_3_^2-^ bending (Rahman et al. 2019), in the uncalcined precipitates of calcium carbonate. The disappearance of the intense band confirmed the decomposition of CaCO_3_ to CaO (Fig. 3) (Erchamo et al. 2021). The intensity of the CaCO_3_ peak was found to decrease, and the peaks around 1450.69 cm^-1^ (Habte et al. 2019) and 1437.67 cm^-1^ (Ngadi et al. 2017) corresponded to the Ca-O bending vibrations. The broad bands in 3500 − 3000 features shown by the nano-catalyst in our study agree with the already reported data in the literature (Jadhav et al. 2022).

**Fig. 4:**
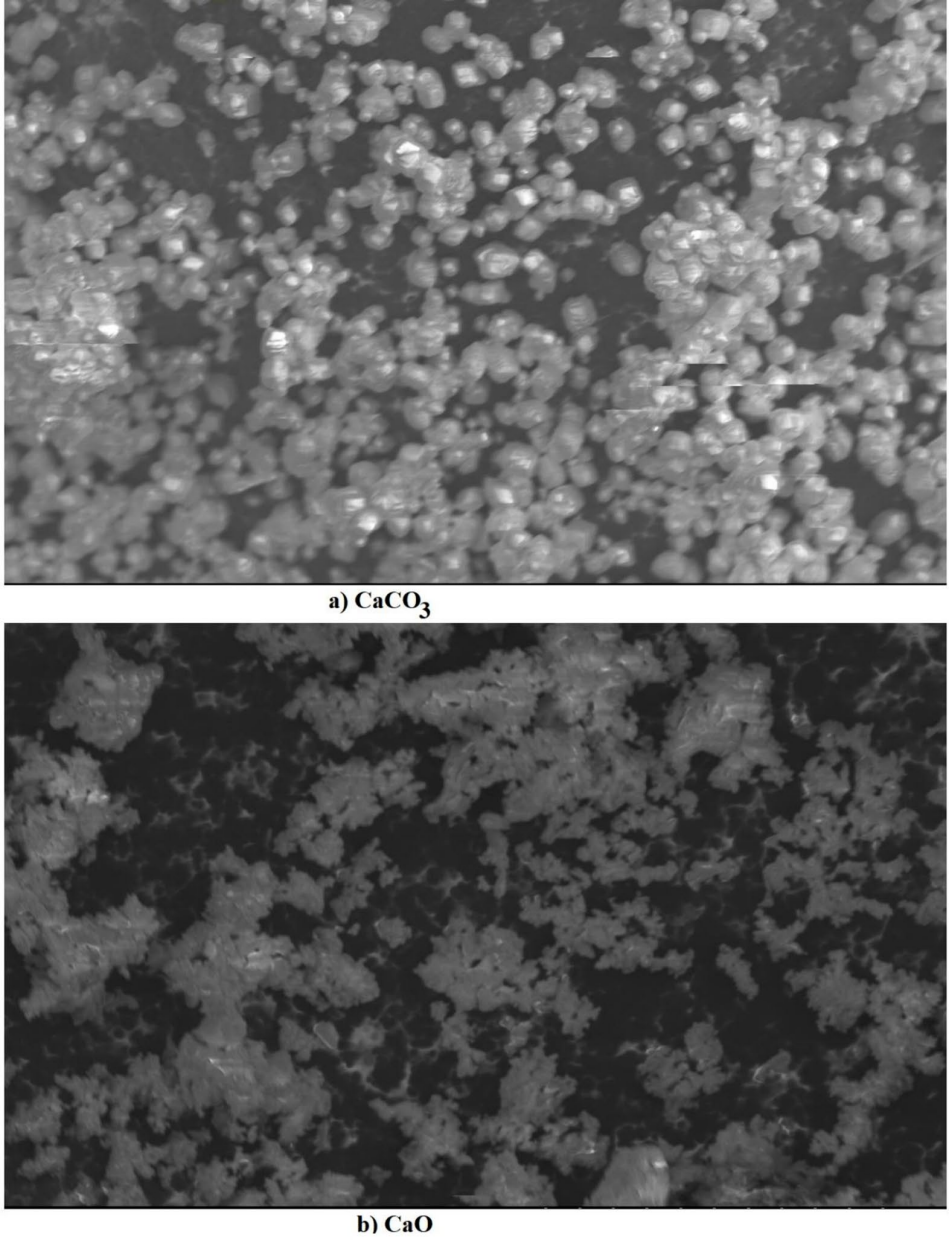
SEM image of precipitated calcium carbonate (**a**) and nano-catalyst (**b**)

The microstructure of uncalcined precipitates and the nano-catalyst was analyzed by SEM. It is a fundamental technique for visualizing the morphology and size of the nanoparticles. SEM images reveal the shape, size distribution, and degree of aggregation of the CaCO3 particles (Ulakpa et al. 2024). It was observed that the microstructure of uncalcined precipitates containing CaCO_3_ with the average particle size ranging around ~ 5.0 μm modified to the porous structure of CaO nanoparticles after calcination, as evident in Fig. 4a and b. The nano-catalyst showed spherical particles with regular morphology, with the average particle size ranging between 70 and 100 nm. Further, these structures were more organized, and there was no evidence of anisotropic development. When the gaseous CO_2_ molecules were released, they left behind regular-shaped particles with decreased particle size, increased surface area, and improved catalytic activity. However, the disintegration of CaCO_3_ to CaO also resulted in forming some agglomerates due to a change in composition upon calcination (Fayyazi et al. 2018).

### Analysis of biodiesel

**Fig. 5:**
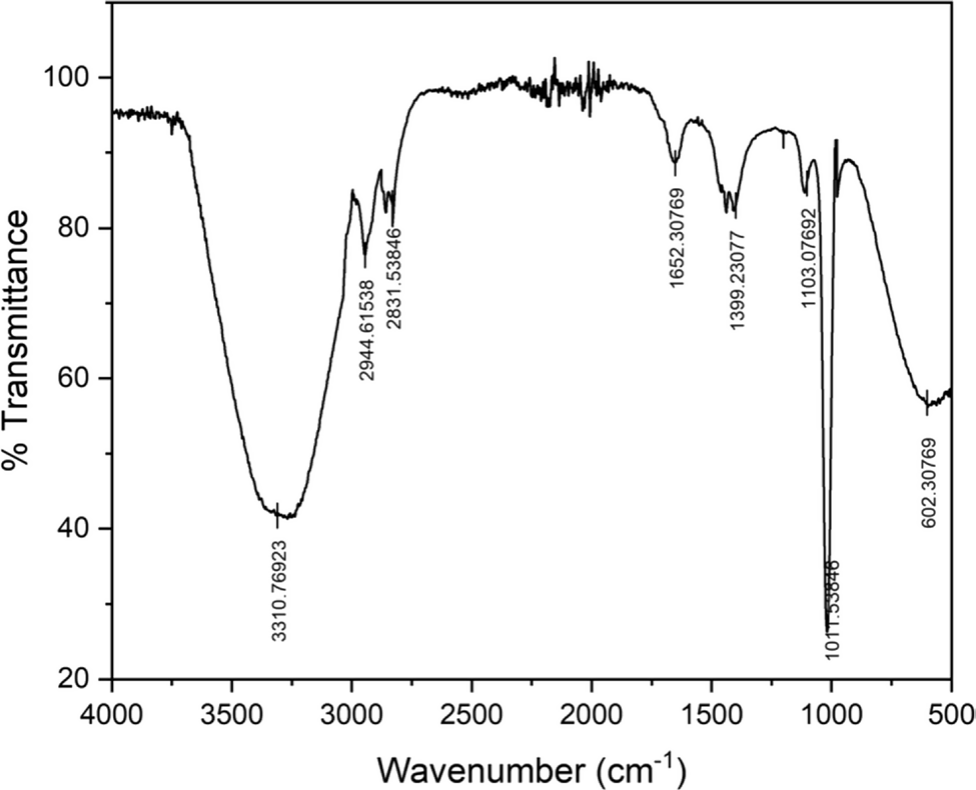
FTIR spectra of biodiesel

The obtained biodiesel has been characterized by various spectrochemical analyses, as discussed. The functional group analysis of biodiesel was carried out by FTIR, as represented in Fig. 5. The peak with the highest intensity at 1652.30 cm^-1^ was assigned to CO stretching in esters (Meena Devi et al. 2015). The bands corresponding to the asymmetric bending of methyl esters in the biodiesel were observed at 1399.23 cm^-1^, while the stretching vibrations of methoxy groups were obtained at 1103.07 and 1011.54 cm^-1^ (Mumtaz et al. 2012). Absorption bands indicated the axial deformation of the CH_2_ bond at 2944.62 and 2831.54 cm^-1^ (Erchamo et al. 2021). The O-H stretching vibrations were observed as a broad band at 3310.77 cm^-1^ (Hangun-Balkir 2016). The band at 602.31 cm^-1^ corresponded to CH_2_ and CH_3_ bending vibrations (Purandaradas et al. 2018). The data coincides with the values observed in the literature for biodiesel synthesis.

**Fig. 6:**
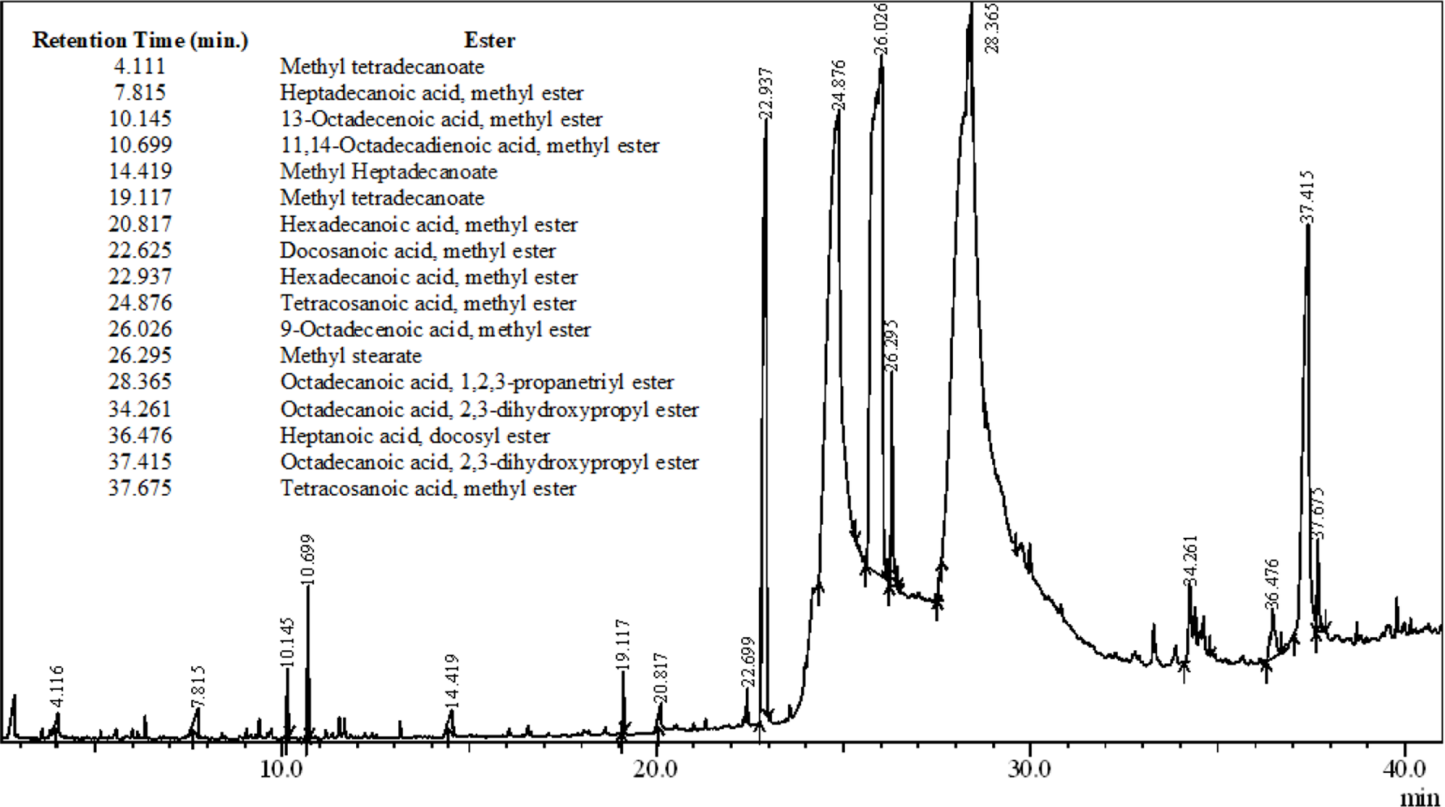
GCMS analysis of biodiesel

The retention time and pattern of mass fragmentation of GCMS analysis recognized the chemical composition of the obtained biodiesel in terms of FAME. Figure 6 provides the composition of the biodiesel produced under optimized conditions and confirms the completion of the reaction with the formation of various esters. The results were consistent with the already reported results (Purandaradas et al. 2018).

The physicochemical properties of biodiesel have been listed in Table 1 with range lying in the permissible standards (Sarkar et al. 2024). Table 2 shows the physicochemical properties of the biodiesel.

**Table 2:** Physicochemical properties of the biodiesel

Property	Values (ASTM) (Erchamo et al. 2021)	Value (Experimental)
Density (At 15^o^C)	880	882 kg/m^3^
Acid value	Max. 0.5	0.43
Iodine value	NS	112 g iodine/100 g
Flash point	100–170	190^o^C
Pour point	-15 to 10	9^o^C
Cloud point	-3-12	-3^o^C
Kinematic viscosity	1.9-6.0	5.31

### Mechanism for the synthesis of biodiesel

Biodiesel production has been achieved through the transesterification of WCO as a feedstock in the presence of methanol and a suitable heterogeneous CaO nano-catalyst. Transesterification involves the reaction of triglycerides in WCO with methanol to produce FAME, the primary components of biodiesel (Ullah et al. 2014). To catalyze the transesterification process efficiently, strong basic sites are required. The use of CaO nano-catalyst for the transesterification of WCO into biodiesel presents a promising approach for sustainable fuel production. CaO, an inexpensive and readily available alkaline catalyst, facilitates the reaction by providing a solid surface for the reaction to occur without dissolving in the reaction mixture. However, understanding the reaction mechanism and the factors influencing its efficiency is crucial. Several studies have explored these aspects, offering insights into the reaction pathways and optimization strategies. The transesterification reaction proceeds in three consecutive steps(Basumatary et al. 2023):

#### Initial interaction

The basic surface sites of CaO nanoparticles attract and adsorb triglyceride molecules. Factors such as surface area and pore structure influence the number of available active sites for triglyceride adsorption (Ulakpa et al. 2024).

#### Nucleophilic attack

Methanol molecules are also adsorbed onto the CaO surface, where the catalyst facilitates proton abstraction, forming the methoxide anion. This anion undergoes a nucleophilic attack on the carbonyl carbon of the ester linkage in the triglyceride. The efficiency of this step depends on reaction temperature, methanol-to-oil molar ratio, and catalyst loading (Yusuff et al. 2024).

#### Ester exchange and glycerol formation

**Fig. 7:**
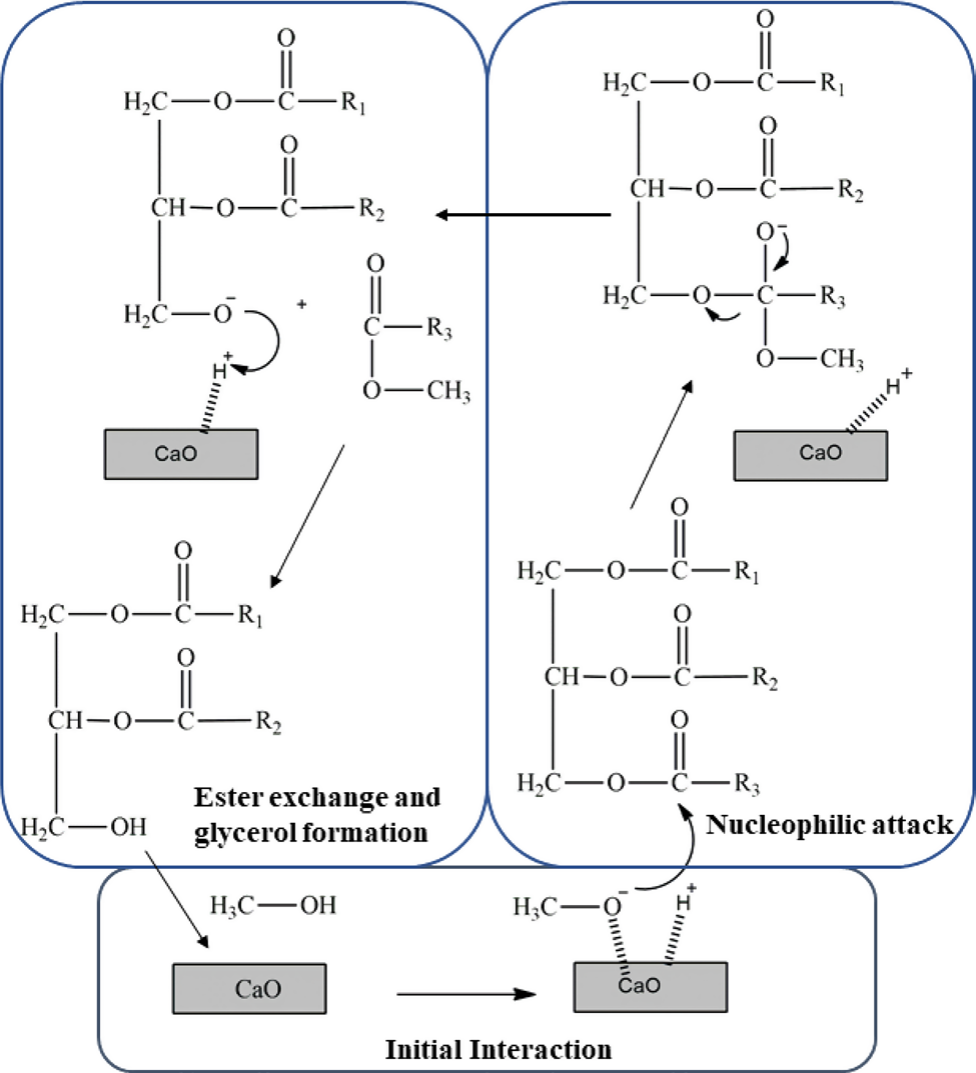
Mechanism for synthesis of biodiesel using CaO nano-catalyst

The nucleophilic attack results in the formation of a tetrahedral intermediate, which subsequently collapses to form a diglyceride and a FAME molecule. The diglyceride anion is stabilized by the abstracted proton and the catalyst is regenerated. The step is repeated with an attack of nucleophiles on all the carbonyl centers of the triglyceride, which will result in the production of three moles of FAME and one mole of glycerol as illustrated in Fig. 7 (Basumatary et al. 2023).

### Impact of operating conditions on biodiesel synthesis

The efficiency and effectiveness of this process are significantly influenced by a complex interplay of operating conditions (Ullah et al. 2014). These critical variables include reaction temperature, the molar ratio of alcohol to oil, the type and concentration of the catalyst employed, and the reaction time. A comprehensive understanding of these parameters is paramount for optimizing biodiesel production, ensuring high yields, maintaining consistent product quality, and ultimately, achieving cost-effectiveness and environmental sustainability (Ullah et al. 2019). With these considerations, transesterification was performed with varying operating conditions, and their impact on the biodiesel yield was analyzed to obtain optimum operating conditions with the best biodiesel yield.

**Fig. 8:**
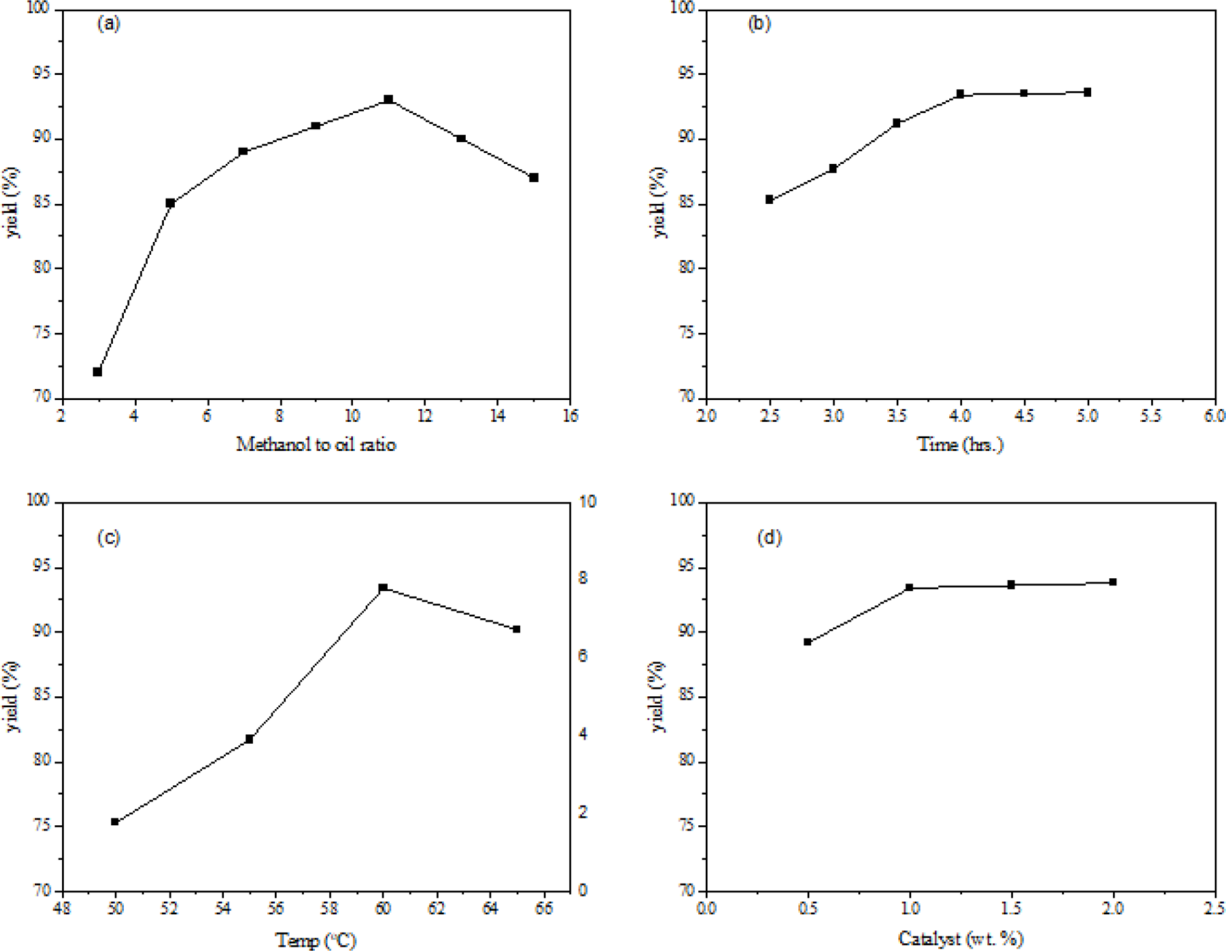
Variation of Biodiesel yield with variation in methanol to oil ratio (**a**), time (**b**), Temperature (**c**) and catalyst wt% (**d**)

The molar ratio of methanol and oil is an important factor affecting the equilibrium and yield of the transesterification reaction. Biodiesel production increases when the methanol-to-oil molar ratio increases. This is because a large concentration of methanol not only increases the amount of methoxy species but also increases the likelihood of effective interactions between the reactants, which increases the conversion rate (Ullah et al. 2024). However, using a very high molar ratio increases the production cost because methanol is a significant expense in biodiesel manufacturing. In addition, extracting unreacted methanol from biodiesel production requires more resources and energy, which makes downstream refining more difficult. The ideal molar ratio should balance between cost reduction and maximum yield. Also, excess methanol has been reported to exhibit a negative impact after a particular ratio (Sodhi et al. 2017). Figure 8a displays the impact of the methanol: oil molar ratio on biodiesel formation. It is evident that the yield of biodiesel increased initially with an increasing molar ratio, up to 11:1. A slight decrease was found beyond 11:1. The literature reports that exceedingly high concentration of methanol not only interferes with the parting of glycerol owing to an enhanced solubility but also causes the equilibrium to shift backward resulting in a reduced yield of biodiesel (Ngadi et al. 2017).

A fundamental factor influencing the degree of triglyceride conversion to biodiesel is contact time. Several elements define the ideal contact time: temperature, type and concentration of the catalyst, molar ratio of reactants, and mixing intensity (Ullah et al. 2017). Determining the ideal reaction time is matching the general process efficiency with the intended degree of conversion. Transesterification is a reversible process that needs more time to attain equilibrium. As the reaction moves toward equilibrium, longer reaction periods usually translate into better biodiesel yields. The longer contact time increases the interaction between methanol and oil at the active sites of the catalyst, thereby enhancing catalytic activity and resulting in higher yields of biodiesel (Sodhi et al. 2017). Hence, during the initial runs, the reaction was allowed to proceed for 2.5 h. The reaction time effect was further increased up to 5.00 h. Figure 8b shows that an increase in contact time of up to 4 h. enhanced the yield. Extending response time outside the ideal point, however, does not proportionately raise the yield and may result in higher energy consumption and manufacturing costs. The hydrolysis of methyl esters may also interfere with the principal reaction causing a negligible increase as evident in Fig. 7b after 4.0 h (Erchamo et al. 2021).

The rate and extent of the transesterification reaction are strongly influenced by reaction temperature (Ullah et al. 2019). Elevated temperatures usually accelerate reaction kinetics by raising the kinetic energy of the interacting molecules, therefore enabling the overcoming of activation energy barriers and supporting more frequent and intense contact between reactants. Introducing the heterogeneous catalyst to the reaction generates a three-phase (oil-methanol-catalyst) system with its interface accommodating the reaction (Sodhi et al. 2017). The reaction temperature is important in biodiesel synthesis because it reduces oil viscosity and boosts biodiesel production as mass transfer resistance is suppressed. Faster reaction rates and, within a given range, increased biodiesel yield follow from this action. Figure 7c displays the impact of variation of reaction temperature from 50 to 65 °C on biodiesel yield. Still, this favorable relationship is not unqualified as evident in Fig. 8c with an increase of biodiesel yield till 60^o^C followed by a decrease afterward. Litearture reports that a reaction temperature higher than 60^o^C also results in a decreased yield of biodiesel owing to the evaporation of methanol (Sarkar et al. 2024). Extremely high temperatures can also cause various negative side reactions, most famously saponification—the process by which triglycerides react with an alkaline catalyst to produce soap. Saponification reduces biodiesel yield, brings contaminants into the product, and requires extra, energy-intensive purifying operations to eliminate the soap (Ullah et al. 2024). Thus, the studies were continued at 60^o^C, considered as the optimal reaction temperature.

The choice of catalyst and its concentration are critical factors affecting the rate, yield, and selectivity of the transesterification reaction (Ullah et al. 2017). Increasing catalyst concentration generally accelerates the reaction rate up to a certain point. The catalyst concentration significantly affects biodiesel production because it increases the number of active basic sites, which increases biodiesel formation (Erchamo et al. 2021). The nano-catalyst has active basic sites attributed to the transformation of methanol into a methoxide group for attacking the carbonyl moiety of oil (Yusuff et al. 2024). The impact of increasing catalyst concentration on biodiesel production is evident in Fig. 8d. The biodiesel yield increased as the catalyst dosage varied from 0.5 to 2.0%. However, the catalyst amount was selected as 1.0% for optimum conversion. An increase of catalyst amount after 1.5% (w/w) was found to not greatly affect the yield, probably because the viscosity of the reaction mixture increases and active sites are loaded with products (Ngadi et al. 2017). Beyond this optimal concentration, however, further increases can lead to increased soap formation or other side reactions, ultimately lowering the biodiesel yield and increasing purification challenges (Ullah et al. 2014). In our current study, the optimal methanol: oil molar ratio was established as 11:1 at a nano-catalyst concentration of 1.0% (w/w), as confirmed by an optimal yield of 93.4% at 60^o^C.

Heterogeneous catalysts, such as CaO remain in a separate phase during the reaction, simplifying catalyst separation and enabling reuse. The efficient recovery and purification of CaO nanoparticles after the transesterification reaction are essential prerequisites for their reuse. It was possible to reuse the catalyst for up to five transesterification cycles in consistence with the earlier reported work (Obadiah et al. 2012). The reusability of CaO nanoparticles is influenced by several factors that contribute to catalyst deactivation. The active sites on the CaO nanoparticles may leach into the reaction mixture, leading to a gradual loss of catalytic activity. These impurities left behind by during recovery can also adsorb onto the active sites, blocking access for the reactants and inhibiting the transesterification reaction (Ulakpa et al. 2024). The aggregation of CaO nanoparticles can reduce their effective surface area and hinder reactant access to the active sites. Aggregation is a common phenomenon for nanoparticles, particularly in liquid media, and can significantly impact catalytic performance. The accumulation of carbonaceous deposits on the surface of the CaO nanoparticles can also lead to catalyst deactivation. These deposits can block active sites and reduce the catalyst’s effectiveness. Table 3 provides a comparative account of the effectiveness of the green synthesized caO nano-catalyst for the transesterification of WCO (Erchamo et al. 2021).

**Table 3:** Performance of varied synthesized CaO nanoparticles in biodiesel synthesis

Type of nano-catalyst	Feedstock	Reaction Temperature (^o^C)	Contact time (hrs.)	Metahnol-Oil Ratio	Nano-catalyst dosage	Biodiesel Yield (%)	References
CaO nanoparticles derived by Sol-gel method	Non-edible seed oil from Argemone mexicana L.	55	1.5	9:1	15 mg	96	(Jan et al. 2024)
Chicken eggshells derived CaO nanoparticles	Waste Soyabean oil	-	2.2	7:1	6.0 wt%	91.4	(Ayoola et al. 2019)
Chicken eggshells derived CaO nanoparticles	Waste palm and sunflower oil	60	2	12:1	2.5 wt%	94	(Erchamo et al. 2021)
Snail shell derived CaO nanoparticles	Jatropa Oil	55	0.5	6:1	1.4 wt%	96.73	(Ulakpa et al. 2024)
Eggshells derived CaO nanoparticles	Waste frying oil	50	3	10:1	0.75 wt%	90.81	(Yusuff et al. 2024)
Green synthesized CaO	Waste soyabean oil	60	4	11.1	1.0 wt%	93.4	This work

## Conclusion

The present study provides an easy and economical pathway to utilize the two common waste products, orange peels, and waste cooking oil, to synthesize value-based products. In this context, calcium oxide nanoparticles were obtained via a green process utilizing orange peel extract. Phase–I of the synthesis yielded microparticles of calcium carbonate in calcite, aragonite, and vaterite phases while phase-II produced the porous nanoparticles of CaO with particle sizes ranging between 70 and 100 nm. By varying reaction parameters, these nanoparticles were effectively employed as heterogeneous nano-catalyst in the transesterification process. The study confirmed an optimal yield of 93.4% under optimized reaction conditions of 60^o^C, 11:1 as methanol to oil ratio after 4 h. and using a catalyst concentration of 1.0%. The green synthesized nano-catalyst was found to be effective in reusability after recycling. Our current study provides a pathway to utilize the waste material economically to obtain biodiesel that is an alternative to exhausting natural petroleum resources leading to sustainable waste valorization.

## Data Availability

The datasets generated during and analyzed during the current study are available from the corresponding author upon reasonable request.
